# Thermally triggered polyrotaxane translational motion helps proton transfer

**DOI:** 10.1038/s41467-018-04733-4

**Published:** 2018-06-12

**Authors:** Xiaolin Ge, Yubin He, Xian Liang, Liang Wu, Yuan Zhu, Zhengjin Yang, Min Hu, Tongwen Xu

**Affiliations:** CAS Key Laboratory of Soft Matter Chemistry, Collaborative Innovation Center of Chemistry for Energy Materials, School of Chemistry and Materials Science, University of Science and Technology of China, 96 Jinzhai Road, 230026 Hefei, Anhui China

## Abstract

Synthetic polyelectrolytes, capable of fast transporting protons, represent a challenging target for membrane engineering in so many fields, for example, fuel cells, redox flow batteries, etc. Inspired by the fast advance in molecular machines, here we report a rotaxane based polymer entity assembled via host–guest interaction and prove that by exploiting the thermally triggered translational motion (although not in a controlled manner) of mechanically bonded rotaxane, exceptionally fast proton transfer can be fulfilled at an external thermal input. The relative motion of the sulfonated axle to the ring in rotaxane happens at ~60 °C in our cases and because of that a proton conductivity (indicating proton transfer rate) of 260.2 mS cm^−1^, which is much higher than that in the state-of-the-art Nafion, is obtained at a relatively low ion-exchange capacity (representing the amount of proton transfer groups) of 0.73 mmol g^−1^.

## Abstract (web summary)

Proton exchange is critical in many applications, such as in conductive proton exchange membranes, but achieving fast proton exchange still remains a challenge. Here the authors report fast proton exchange in a rotaxane based polymer by exploiting thermally triggered translational motion of the mechanically bonded rotaxane.

## Introduction

Fast proton transfer remains a big challenge in so many fields^1,2^. For instance, highly conductive proton exchange membranes are vital in bumping up the power output of polymer electrolyte based H_2_/O_2_ fuel cell (a typical example for clean energy production)^3,4^. Fast proton transfer is also considered critical in vanadium flow batteries or organic redox flow batteries (representing the most recent advance in energy storage)^5^. Conventionally, protons are transferred via negatively charged carriers (for instance, sulfonates) in either polymeric electrolytes or proton exchange membranes and these carriers are covalently bonded to the polymer backbone^6,7^, which has limited mobility due to large molecular weight. The limited mobility thereby results in low proton transfer rate, i.e., low proton conductivity and it remains the bottleneck to further enhance the efficiency of many energy conversion and energy storage processes, in which proton transfer is involved^8,9^.

The external stimuli triggered motion in a molecular machine motivates us and sheds lights on how to further increase proton transfer rate/efficiency. A molecular machine, defined as an assembly of a distinct number of molecular components that are designed to perform machinelike movements as a result of an appropriate external stimulation^10,11^, has been successfully demonstrated and built, based on topological entanglement (mechanical bonds)^12–14^ or isomerisable unsaturated bonds^15^. Controlled translational/rotational motion and controlled unidirectional rotation in such ingenious design represent the most intricate aspects in making a macroscopic prototype^16,17^. Yet inspirations from such delicate design could lead to a giant leap forward in many fields.

We proved herein that by introducing the thermally triggered translational motion (although not in a controlled manner) of mechanically bonded rotaxane in a polymeric entity, exceptionally fast proton transfer could be attained at an external thermal supply. We implemented rotaxanes (based on a ring threaded over an axle with stoppers at both ends) in a polymer and proved that as a result of a thermal input (an increase in temperature), the relatively translational motion of the ring to the axle happens, which was however confined by the stoppers. The axle was modified with sulfonates (thereby negatively charged), which acted as proton transfer carrier. As a consequence, an proton conductivity (indicating proton transfer rate) of 260.2 mS cm^−1^, which is much higher than that in the state-of-the-art Nafion (Dupont, USA, $300.00 m^−2^, http://www.nafionstore.com/), was obtained at a relatively low ion-exchange capacity of 0.73 mmol g^−1^ (representing the amount of proton transfer groups). The knowledge we learned and the results we obtained here could benefit so many fields, where proton transfer is involved, for example, fuel cells^18^, redox flow batteries^19^, etc.

## Results

### Assembling a rotaxane based polymer entity

**Fig. 1:**
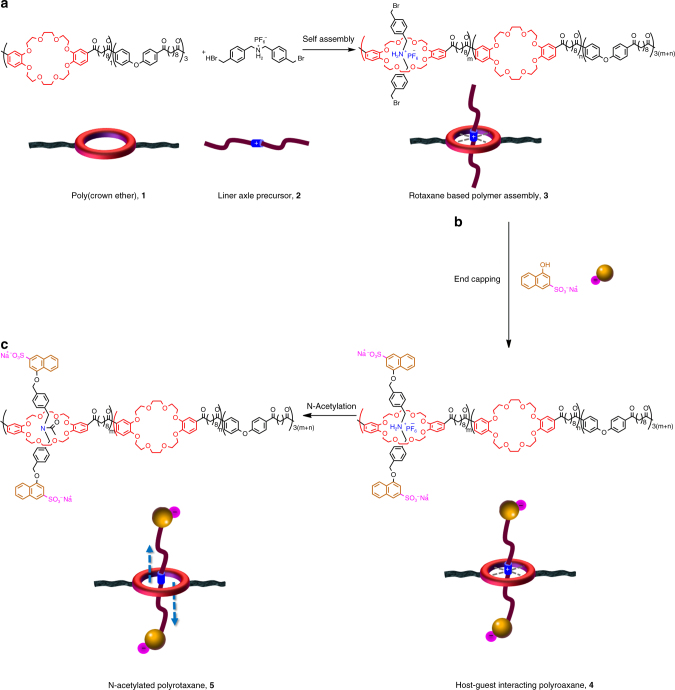
Synthesis of a rotaxane based polymer entity. **a** Poly(Crown ether), **1**, was synthesized via polyacylation and the host–guest interaction between **1** and **2** results in rotaxane based polymer assembly, **3**. **b** End-capping of **3** was performed to prevent the diffusion loss of movable axles, leading to **4**. **c** N-acetylation of **4** leads to the target polymer, **5**, having axles with enhanced mobility. For synthetic details and characterizations, see Supplementary Figs. 1–13

As opposed to the circumstance in a conventional proton transfer polymer/membrane in which the proton transfer carriers (negatively charged functional groups) are fixed to the polymer backbone, we included rotaxanes, which could provide translational motion when the thermal stimulus is applied, to further prompt proton transfer rate in such polymers/membranes. To construct such assembly (Fig. 1a), we firstly synthesized a poly (crown ether) backbone (**1**) via polyacylation (Supplementary Figs. 1 and 2) and a linear axle precursor, **2**. Synthesis and characterizations of the linear axle precursor were described in detail in the Methods (see also Supplementary Figs. 3–8). The rotaxane based polymer entity, **4**, was then assembled by threading the linear axles into the poly (crown ether) backbone via host–guest interaction between the electron-rich crown ether donor and electron-poor *sec-*ammonium axles (resulting **3**), both ends of which were then end-capped by a sulfonated stopper (negatively charged), the bulky 1-naphthol-3-sulfonic acid sodium salt (Fig. 1b and Supplementary Figs. 9 and 10). The resulting negatively charged axles could serve as potential proton transfer carriers. However, the axles might be trapped in the ring because of the strong electrostatic host–guest interaction, which requires more energy input to break. More freedom is then given to the linear axles by breaking the electrostatic host–guest interaction via N-acetylation (resulting **5**, Fig. 1c and Supplementary Figs. 12 and 13), enabling the axles with enhanced moving capability^20^.

**Fig. 2:**
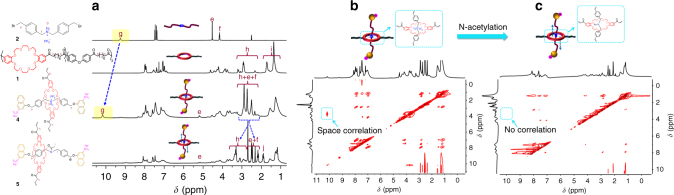
Probing host–guest interaction in the rotaxane based polymer assembly. **a** ^1^H-NMR spectra (from top to bottom) of linear axles precursor **2**, (400 MHz, DMSO-d_6_, 298 K), the poly(crown ether) **1**, (400 MHz, CDCl_3_, 298 K), polyrotaxane **4** (assembled from the **1** and **2** via host–guest interaction, 400 MHz, DMSO-d_6_, 298 K) and N-acetylated polyrotaxane, **5** (400 MHz, DMSO-d_6_, 298 K). **b** NOSEY spectrum of polyrotaxane **4** from the assembly of **1** and **2** (400 MHz, DMSO-d_6_, 298 K). **c** NOSEY spectrum of N-acetylated polyrotaxane, **5** (400 MHz, DMSO-d_6_, 298 K)

Elemental analysis results (Supplementary Table 1) confirmed that 79.9% (molar) of the crown ether groups in the polymer backbone was threaded by the linear axles. The successful threading-end capping was confirmed via ^1^H nuclear magnetic resonance (NMR) spectroscopy. As shown in Fig. 2a, typical proton signal of the *sec-*ammonium group in the axle shifts downfield because of the strong host–guest interaction and it happens when the linear axle was surrounded by the macrocyclic crown ether. As further confirmed in the nuclear overhauser effect spectroscopy (NOESY, Fig. 2b), proton signals from the macrocyclic crown ether show strong spatial correlation with signals from protons of the *sec*-ammonium group in the axles, as highlighted by the rectangle.

### Thermally triggered motion of rotaxanes

Differential scanning calorimetry (DSC) curve suggests that the host–guest interaction between *sec*-ammonium moieties from the axles and the crown ether rings is sufficiently strong. A higher activation energy is required to disrupt the host–guest interaction (a distinct endothermic peak over the temperature range of 65–75 °C, Supplementary Fig. 14). To render the axles with improved mobility, N-acetylation of *sec*-ammonium moieties was performed (Supplementary Figs. 12 and 13)^21^. And we observed the disappear of the *sec*-ammonium proton signal and a new proton signal of the acetyl group at *δ* = 2.5 ppm appears (Fig. 2a and Supplementary Fig. 13) in the ^1^H NMR spectrum of the N-acetylated polyrotaxane. We proposed that by eliminating the positive charge in the center ammonium groups via N-acetylation, the host–guest interaction could be weakened, leading to enhanced axle mobility. As confirmed in the space correlation spectrum (NOESY), no strong host–guest interaction signal could be observed (as highlighted in the rectangle, Fig. 2c), suggesting that the axles are moving. This moving mechanism helps us better understand the ^1^H-NMR spectral changes before and after N-acetylation of polyrotaxane. Before N-acetylation, proton signals from the rotaxane moiety overlap in the range of 3.3–2.3 ppm. After N-acetylation, they shift significantly because of the shielding/deshielding effect of the crown ether ring, which is not flat and folds like the letter C^22^. Partial of proton signals from the axles, which were trapped in the crown ether ring, is shielded, leading to upfield shifts, while others are de-shielded (shifting downfield).

We assumed that the movable nature of the proton carrier, the negatively charged axles, would facilitate proton transfer. To demonstrate such capability, we converted the polyrotaxane polymers into membrane shape, which is commonly referred to as a proton exchange membrane, by solution casting the N-methyl-2-pyrrolidone (NMP, the solvent) solution of **5** (10 wt%) in a clean PTFE petri dish and a transparent membrane (with thickness of 90 ± 7.5 μm, mean ± SD (standard deviation), from five repeated measurements) was obtained after complete solvent evaporation. The counter-ions of the movable axles were then converted to H^+^ in 1 M aqueous HCl at 25 °C for 24 h prior to further characterizations. It should be noted that the acetylated *sec*-ammonium groups would not be affected by the ion exchange treatment. Titrations of the H^+^ ions inside the membrane suggest a relatively low ion-exchange capacity (IEC, the amount of functional group content), which is in good accordance with elemental analysis results (0.73 mmol g^−1^, Supplementary Table 2). Water uptake value (measured by the increased weight of dry membrane samples after immersion in DI water at certain temperature for 24 h) indicates that, in average, each sulfonated axle is surrounded by 14 water molecules at 30 °C or by 58 water molecules at 60 °C (Table 1). Despite the amount of water adsorbed, mechanical strength of the as-prepared polyrotaxane membrane is acceptable in the wet state for potential applications (Table 1 and Supplementary Table 3).

**Table 1:** Properties of the polyrotaxane proton exchange membranes

Membrane	Grating ratio (GR)^a^	Ion exchange capacity (IEC)	Water uptake (WU)		*λ* (water content ratio)^**b**^		Mechanical properties (60 °C, wet)^c^	
			30 °C	60 °C	30 °C	60 °C	**TS** (MPa)	**Eb** (%)
Polyrotaxane	79.9%	0.73 (mmol g^−1^)	18.2%	76.6%	14	58	12.01	20.69

^a^Grafting ratio, calculated from the content of the sulfonated axles and crown ether moieties based on the elemental analysis results

^b^*λ*, Water content ratio, a molar ratio of water molecules to the sulfonated axles, calculated from WU and IEC

^c^TS, tensile strength measured in the stress–strain curve; Eb, elongation at break

**Fig. 3:**
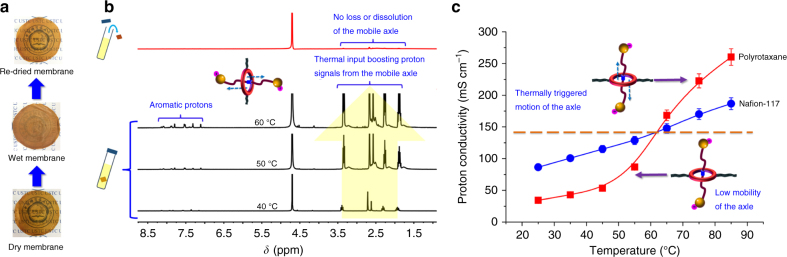
Appearance, thermal responsive ^1^H-NMR and temperature-dependent proton conductivity of the polyrotaxane membrane. **a** Digital photos of polyrotaxane membrane samples, showing the thermal responsive transparency change in membrane appearance, from bottom to top: dry membrane sample, hydrated membrane sample (treated in 60 °C water for 2 h) and re-dried membrane sample. **b** ^1^H-NMR spectra (400 MHz, D_2_O) of membrane samples immersed in D_2_O as the temperature was increased from 40 to 60 °C, and spectrum recorded from the same NMR tube after removing the membrane sample (top spectrum). **c** Proton conductivities of polyrotaxane membrane and the benchmark Nafion-117 as a function of temperature. The error bars represent the s.d. from three independent measurements

The thermally-triggered translational motion of the rotaxanes in the as-prepared membrane was observed in two distinct experiments. We first observed the transparency change of the as-prepared polyrotaxane membrane when the thermal stimulus was applied. As shown in Fig. 3a, the dry membrane samples were transparent in the first place, which gradually became opaque when they were treated in hot water for 2 h (60 °C). And the transparency of the membrane samples was recovered after the samples were dried. We have confirmed that disappear and recovery of transparency in these membrane samples is repeatable. Noting that treating the membrane samples in room temperature water does not cause transparency change and only could slight water swelling be found. We have proved via NMR that the transparency change we observed is not caused by the loss or dissolution of the movable axles since no signals were detected in D_2_O when the membrane sample was removed (top spectrum in Fig. 3b). We, therefore, conclude that the transparency change in membrane samples is caused by the thermally-triggered motion of rotaxanes. And the thermally triggered translational motion of rotaxanes in the membrane samples was also reflected in the temperature dependent ^1^H NMR spectra (Fig. 3b). Although in the membrane state, we have detected the proton signals of the hydrated axles in the 40 °C ^1^H NMR spectrum, which is extremely weak as expected. As the temperature continues to rise, from 50 to 60 °C, the corresponding signals get stronger and stronger, because sufficient energy is provided to activate fast axle movements.

### Accelerated proton transfer

Because of the thermally triggered motion of rotaxanes, the negatively charged axles (bearing sulfonated functional groups as proton carrier) are now movable at a thermal stimulus. One benefit from the movable proton carriers (axles) in a polyrotaxane membrane is the accelerated proton transfer. We have measured the proton conductivity (an indicator of proton transfer rate, was measured under 100% humidity and various temperature using a four-electrode AC impedance method) of the as-prepared polyrotaxane membrane samples and that of the benchmark Nafion-117 membrane (a commercially available, state-of-the-art proton exchange membrane) (Fig. 3c). As shown, the conductivity-temperature curve of Nafion-117 membrane shows almost linearly increasing trend, rising from 86.5 mS cm^−1^ at 25 °C to 186.7 mS cm^−1^ at 85 °C and this trend is commonly observed among other proton exchange membranes. Nevertheless, the conductivity-temperature curve of the as-prepared polyrotaxane membrane shows a non-linear increasing trend, divided into two distinct segments. The proton conductivity increases slowly in the temperature range from 25 to 55 °C, and the proton conductivity at 55 °C is 86.7 mS cm^−1^, which is much lower than that of Nafion-117 (128.7 mS cm^−1^). After a critical temperature in between 55 and 65 °C (which is assumed to be ~60 °C according to our NMR studies in previous sections), the proton conductivity is sharply increased and rockets up to 260.2 mS cm^−1^ at 85 °C, which is 1.4 times that of Nafion-117 (186.7 mS cm^−1^). Further comparison with other reported hydrocarbon-based PEMs also suggests a more efficient proton transfer in the polyrotaxane membrane at elevated temperatures (Supplementary Fig. 18). It is attributed to the thermally-triggered translational motion of rotaxanes, activating movable proton carriers in the polyrotaxane membranes. At temperatures lower than ~60 °C, the external energy input (thermal stimulus) is not strong enough to power the motion of rotaxanes. And we have demonstrated that the proton conductivity response to operating temperature can be repeatedly observed in the forward and backward temperature scan (Supplementary Fig. 17).

**Fig. 4:**
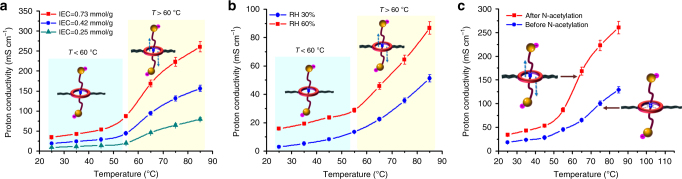
Proton conductivity of polyrotaxane membranes. **a** Proton conductivity of polyrotaxane membranes with varied IEC values as a function of temperature. **b** Proton conductivity of the polyrotaxane membrane (IEC = 0.73 mmol g^−1^) as a function of temperature at 30% RH and 60% RH. **c** Proton conductivity of the polyrotaxane membrane (IEC = 0.73 mmol g^−1^) before and after N-acetylation as a function of temperature. The error bars represent the s.d. from three independent measurements

In a control experiment, we varied the IEC values of the polyrotaxane membrane and we observed the same trend in proton conductivity change over operating temperature, suggesting the thermally-triggered motion of the linear axles is IEC-independent (Fig. 4a). Proton conductivity measurements under different humidified conditions prove that the increased proton conductivity at higher temperature is not caused by the change in membrane water uptake (Supplementary Figs. 15 and 16). In a less humidified conditions, for instance, 30% RH or 60% RH, we witnessed the same thermal responsive behavior of the polyrotaxane membrane, despite the fact that such behavior is more obvious as humidity rises (Fig. 4b). However, the role of adsorbed water molecules on the thermally triggered motion of rotaxanes remains unclear since we currently do not have the facility to measure the anhydrous proton conductivity of the polyrotaxane membranes, yet we do believe further research in this direction would give us more information on the accelerated proton transfer mechanism. To exemplify the importance of the translational motion of the linear axles in facilitating proton transfer, we fabricated a control membrane, the linear axles of which were strongly entrapped in the crown ether ring via host–guest interaction. N-acetylation of the control membrane renders the linear axles with enhanced mobility, resulting in the polyrotaxane membrane we previously discussed. Proton conductivity measurements at varied temperature demonstrate that only by freeing the linear axles, could accelerated proton transfer be obtained at external thermal stimuli (Fig. 4c). It should be further noted that replacing the linear axles with K^+^ results a membrane that is not capable of transferring protons (Supplementary Fig. 11).

## Discussion

In summary, we have assembled a rotaxane based polymer entity via host–guest interaction and demonstrated that at an external thermal stimulus, translational motion of the axles to the rings in rotaxane moieties happens. By exploiting the translational motion of rotaxanes, movable proton carriers (the negatively charged axles) can therefore be implemented. We found that the thermally triggered movement of proton carriers help proton transfer at a much faster rate than that in traditional proton exchange membranes and this striking feature would benefit various fields in energy conversion and energy storage process where proton transfer is involved.

## Methods

### Chemicals

Hydrobromic acid (HBr) and magnesium sulfate (MgSO_4_) were purchased from Sinopharm Chemical Reagent Co., Ltd. Methanesulfonic acid (98%), diphenyl ether (99%), sebacic acid (98%), methyl-4-formylbenzoate (99%), ammonium hexafluorophosphate (98%), and methyl-4-(aminomethyl)benzoate hydrochloride were purchased from Shanghai Energy-Chemical Co. Ltd. Dibenzo-24-crown-8 and 1-naphthol-3-sulfonic acid sodium salt were purchased from Tokyo Chemical Industry (TCI). Lithium aluminum hydride (LiAlH_4_) (97%), phosphorus pentoxide (P_2_O_5_) and sodium sulfate decahydrate were purchased from Aladdin Industrial Corporation, China. All reagents were used as received.

### Synthesis of graft polyrotaxane and preparation of membrane

The synthesis procedures used to prepare **1**, **2c**, **2d**, **2**, **4**, and **5** are shown in Supplementary Figs. 1–13. Analytical thin-layer chromatography (TLC) was performed on glass sheets pre-coated with the silica gel 60-F254. The ^1^H NMR spectra are recorded in ppm.

Synthesis of compound **1**^23^. Dibenzo-24-crown-8 (1 mmol), sebacic acid (4 mmol) and diphenyl ether (3 mmol) were dissolved in Eaton’s reagent (12.8 mL), and the mixture was heated to 40 °C and stirred for 24 h. After the reaction was stirred for 24 h, it was poured into water. The slightly yellow fibers that formed were filtered off and washed with water. After the fibers were dried, compound **1d** was obtained as a white fiber-like polymer, **2a** (98%). ^1^H NMR (400 MHz, CDCl_3_, 298 K) *δ* 7.97 (dd, *J* = 19.5, 8.1 Hz, 8H), 7.54 (td, *J* = 17.4, 16.3, 6.8 Hz, 4H), 7.40 (t, *J* = 7.9 Hz, 3H), 7.20 (t, *J* = 7.3 Hz, 2H), 7.07 (dd, *J* = 8.6, 3.8 Hz, 8H), 7.03–6.97 (m, 2H), 6.90 (dd, *J* = 8.8, 4.7 Hz, 2H), 4.74–4.56 (m, 2H), 4.40–4.12 (m, 12H), 3.94–3.70 (m, 10H), 3.20 (d, *J* = 4.4 Hz, 3H), 3.07 (t, *J* = 5.0 Hz, 1H), 3.03–2.79 (m, 12H), 2.33 (t, *J* = 7.4 Hz, 2H), 1.88–1.62 (m, 14H), 1.36 (s, 32H).

Synthesis of compound **2c**. Compound **2a** (1 mmol, 1 equiv) and compound **2b** (1 equiv) were dissolved in dry toluene (60 mL), and the mixture was heated under reflux in an argon atmosphere for 24 h using a Dean–Stark apparatus. After the reaction mixture cooled, the solvents were removed under vacuum, and the remaining components were washed with ethanol (3 × 30 mL). Compound **2c** was isolated as a white solid (90%). ^1^H NMR (400 MHz, CDCl_3_, 2.98 K) *δ* 8.46 (d, *J* = 1.4 Hz, 1H), 8.10 (d, *J* = 8.4 Hz, 2H), 8.03 (d, *J* = 8.3 Hz, 2H), 7.86 (d, *J* = 8.4 Hz, 2H), 7.43 (d, *J* = 8.3 Hz, 2H), 4.90 (d, *J* = 1.3 Hz, 2H), 3.93 (d, *J* = 9.6 Hz, 6H).

Synthesis of compound **2d**. A solution of compound **2c** (1 equiv) in dry THF was cooled to 0 °C, and powdered LiAlH_4_ (6 equiv) was added to the solution over a period of 1 h. The mixture was then warmed to room temperature and heated under reflux in an argon atmosphere overnight. After the reaction was stirred overnight, it was cooled to 0 °C. Sodium sulfate decahydrate (2 equiv) was then carefully added to the flask. The mixture was stirred for 1 h, filtered and washed with THF. The filtrate was collected and dried (with MgSO_4_), and the solvent was removed under vacuum to give compound **2d** as a yellow liquid (80%). ^1^H NMR (400 MHz, DMSO-d_6_, 298 K) *δ* 7.45–7.21 (m, 8H), 5.24 (t, *J* = 5.7 Hz, 2H), 4.52 (d, *J* = 4.8 Hz, 4H), 3.68 (s, 4H), 3.56–3.41 (m, 1H).

Synthesis of compound **2**. Compound **2d** (1 mmol) was dissolved in 30 mL of HBr (48%), and the resulting mixture was heated under reflux for 24 h. After the reaction was stirred for 24 h, it was cooled to room temperature, filtered and washed with water. The white solid was dissolved in a saturated, aqueous NH_4_PF_6_ solution and stirred for 2 h. The reaction mixture was again filtered and washed with water to yield a white solid, compound **2** (82%). ^1^H NMR (400 MHz, DMSO-d_6_, 298 K) *δ* 9.25 (s, 2H), 7.47–7.38 (d, *J* = 7.9 Hz, 8H), 4.53 (s, 4H), 4.15 (t, *J* = 5.7 Hz, 4H).

Synthesis of compound **4**. Compound **1** (1 mmol) was dissolved in chloroform (100 mL), and then, **2** (1.2 equiv) was added to the reaction system. The resulting mixture was stirred at room temperature for 6 h. 1-Naphthol-3-sulfonic acid sodium salt (3 equiv) and K_2_CO_3_ (18 equiv) were dissolved in dry DMF (15 mL), and the resulting mixture was placed under a nitrogen atmosphere at 60 °C for 2 h. Then, this reaction system was cooled to room temperature and we used a syringe filter to quickly remove the unreacted K_2_CO_3_, which is a white solid and added the supernatant to the first reaction system. After the mixture was stirred for 72 h under a nitrogen atmosphere at room temperature, it was poured into diethyl ether and stirred. The yellow fibers that formed were filtered off, washed with diethyl ether and dried under flowing air to give **4** as a yellow, fiber-like polymer. ^1^H NMR (300 MHz, DMSO-d_6_, 298 K) *δ* 10.17 (s, 2H), 8.26–7.74 (m, 20H), 7.71–7.29 (m, 24H), 7.28–6.89 (m, 24H), 5.19 (s, 2H), 4.66 (d, *J* = 72.9 Hz, 4H), 4.48–3.97 (m, 18H), 3.97–3.13 (m, 34H), 2.81 (d, *J* = 47.6 Hz, 40H), 2.56–2.28 (m, 24H), 1.41 (d, *J* = 90.9 Hz, 62H).

Synthesis of compound **4-1**. Compound **1** (1 mmol) was dissolved in chloroform (100 mL) and was stirred at room temperature for 6 h. 1-Naphthol-3-sulfonic acid sodium salt (3 equiv) and K_2_CO_3_ (18 equiv) were dissolved in dry DMF (15 mL), and the resulting mixture was placed under a nitrogen atmosphere at 60 °C for 2 h. The mixture was then cooled to room temperature, to which was added the solution of compound **1**. Whereupon the mixture was stirred for 72 h under a nitrogen atmosphere at room temperature. Similar purification procedure was conducted, and we finally obtained a yellowish fiber-like polymer (polymer **4-1**). With the same casting procedure, we obtained a control membrane. We measured the proton conductivity of the control membrane. Unfortunately, no proton conductivity was detected, suggesting no proton transfer capability if the cavity of the crown ether was occupied by K^+^.

Synthesis of compound **5**^24^. A mixture of compound **4** (1 mmol), Et_3_N (5 mmol), and Ac_2_O (3 mmol) in *N,N*-dimethylformamide (DMF, 0.69 mL) was stirred at 40 °C for 24 h. Then, it was poured into diethyl ether and stirred. The yellow fibers that formed were filtered off, washed with diethyl ether and dried under flowing air to give **5** as a yellow, fiber-like polymer^[3]^. ^1^H NMR (400 MHz, DMSO-d_6_, 298 K) *δ* 8.21–7.77 (m, 20H), 7.68–7.37 (m, 20H), 7.07 (d, *J* = 32.0 Hz, 20H), 5.18 (d, *J* = 18.6 Hz, 2H), 4.64–3.99 (m, 22H), 3.69 (d, *J* = 38.3 Hz, 22H), 3.45–3.23 (m, 30H), 3.11 (q, *J* = 6.8, 6.2 Hz, 10H), 2.92 (s, 12H), 2.70 (s, 18H), 2.48 (d, *J* = 12.7 Hz, 20H), 2.32 (s, 6H), 2.18 (t, *J* = 8.1 Hz, 12H), 1.91 (q, *J* = 7.6 Hz, 12H), 1.65–0.93 (m, 62H).

Preparation of polyrotaxane membrane. Compound **5** was dissolved in NMP to form a 10 wt% casting solution that was cast onto a glass plate. The cast films were then heated at 80 °C to remove the solvent. After the drying process, flexible, transparent, and yellow-tinged PEMs were obtained. All the membranes were fully converted to the H^+^ form via immersion in aqueous HCl (1 mol L^−1^) solution at room temperature for 24 h, and this was followed by thorough washing and storage in sealed sample bottles (full of deionized water). The resulting membrane was treated with 1 M aqueous HCl at 25 °C for 24 h to convert the termini into the H^+^ form for proton transport. This membrane was denoted polyrotaxane membrane.

With the same casting procedure, we obtained a control membrane from compound **4-1**. We measured the proton conductivity of the control membrane. Unfortunately, no proton conductivity was detected, suggesting no proton transfer capability if the cavity of the crown ether was occupied by K^+^.

### Instruments and characterizations

NMR and nuclear overhauser effect spectroscopy (NOESY) spectra were recorded on a Bruker Avance III 400 MHz spectrometer. Chemical shifts are reported in ppm relative to the signals corresponding to the residual non-deuterated protons in NMR solvents (CDCl_3_: *δ* 7.26 ppm, DMSO-d_6_: *δ* 2.53 ppm, D_2_O: *δ* 1.56 ppm).

Differential scanning calorimetry thermograms (DSC) were performed on a DSC Q2000 thermal analysis system (TA Instruments, USA). Sample 4 was initially heated from 20 to 100 °C at a heating rate of 10 °C min^−1^ under a nitrogen atmosphere, held at 100 °C for 3 min, and cooled to 20 °C at a rate of 10 °C min^−1^.

Elemental analysis (EA) was recorded on a VarioELcube analyzer (Elementar Analysensysteme GmbH, Germany). Grafting ratio and ion exchange capacity of the PEM were calculated from the sulfur content.

Ion exchange capacity (IEC) is defined as the theoretical number of sulfonic acid groups per weight of the dry membrane. It was calculated from the sulfur content by EA. For comparing, it was measured by titration method. Typically, the membrane sample was firstly immersed in NaCl aqueous solution (1 M) for 24 h. Then, the membrane was washed with DI water to remove the absorbed NaCl and dried to a constant mass and weighed (denoted as *W*_dry_). Finally, the membrane was soaked in Na_2_SO_4_ aqueous solution (0.5 M) for 24 h to exchange Cl^−^ with SO_4_^2−^. The Cl^−^ ions released from the membrane were then titrated with an AgNO_3_ aqueous solution (0.05 M) using K_2_CrO_4_ as a colorimetric indicator. IEC value is calculated from the amount of AgNO_3_ consumed during titration and the mass of the dry membrane in Cl^−^ form (*W*_dry_) as shown:

1 $${\mathrm{IEC}}\left( {{\mathrm{mmol}}\,{\mathrm{g}}^{ - {\mathrm{1}}}} \right) = \frac{{V\left( {{\mathrm{AgNO}}_3} \right){\mathrm{ \times }}C\left( {{\mathrm{AgNO}}_3} \right)}}{{W_{{\mathrm{dry}}}}}$$

Water uptake (WU) was determined by measuring the weight changes in polyrotaxane before and after hydration. Membrane samples (4 cm × 1 cm) were weighed after being dried at 80 °C under vacuum for 12 h and, then, immersed in deionized water at 60 °C for 24 h. The hydrated samples were then wiped with tissue paper to remove excess surface water, and their mass was quickly measured. WU was calculated as follows: by comparing the contents of the ammonium axles and crown ether moieties on the basis of the elemental analysis results

2 $${\mathrm{WU}}\left( \% \right) = {\mathrm{100\% \times }}\left( {W_{{\mathrm{wet}}} - W_{{\mathrm{dry}}}} \right)/W_{{\mathrm{dry}}},$$

where *W*_wet_ and *W*_dry_ are the mass of the hydrated and dry PEM sample, respectively.

Water content (*λ*) was calculated from the values of IEC and WU. It was defined as a mole ratio of water to sulfonic acid sites.

Proton conductivity was measured using a standard four-electrode AC impedance technique^25^. The measuring cell (Teflon) had two outer current-carrying electrodes (stainless steel, flat, 2 cm apart) and two inner potential-sensing electrodes (platinum wire, 1 cm apart). The membrane (4 cm × 1 cm) in the H^+^ form was mounted onto the cell, which was then placed inside an oven with controlled temperature and humidity. The impedance was determined using an autolab PGSTAT 30 (Eco Chemie, Netherland) in the galvanostatic mode with an AC current amplitude of 0.1 mA over a frequency range from 100 kHz to 50 Hz. Using a Bode-type plot, the frequency region in which the impedance was constant was determined, and the corresponding resistance was obtained from a Nyquist plot. The proton conductivity (*σ*) along the membrane was calculated according to the following equation:

3 $$\sigma = L/RWD,$$

where *R* is the obtained membrane resistance, *L* is the distance between the potential-sensing electrodes, and *W* and *D* are the width and thickness of the membrane under the test conditions, respectively.

### Data availability

The authors declare that all data supporting the findings of this study are available within the paper and its supplementary information files. They are also available from the corresponding authors upon reasonable request.

## Electronic supplementary material

Supplementary Information
