# Supplementary material for: Thermally triggered polyrotaxane translational motion helps proton transfer
Source: Nat Commun. 2018 Jun 12;9:2297. doi: 10.1038/s41467-018-04733-4 (PMC5997710; doi:10.1038/s41467-018-04733-4)
Supplement: Supplementary file 1 — Supplementary Information [file 41467_2018_4733_MOESM1_ESM.pdf]

Supplementary Information for

Thermally triggered polyrotaxane translational motion helps proton transfer

Ge et al.

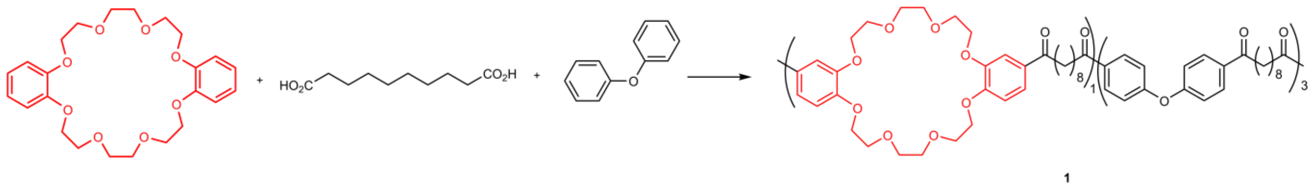

Supplementary Figure 1 Synthesis of **1**.

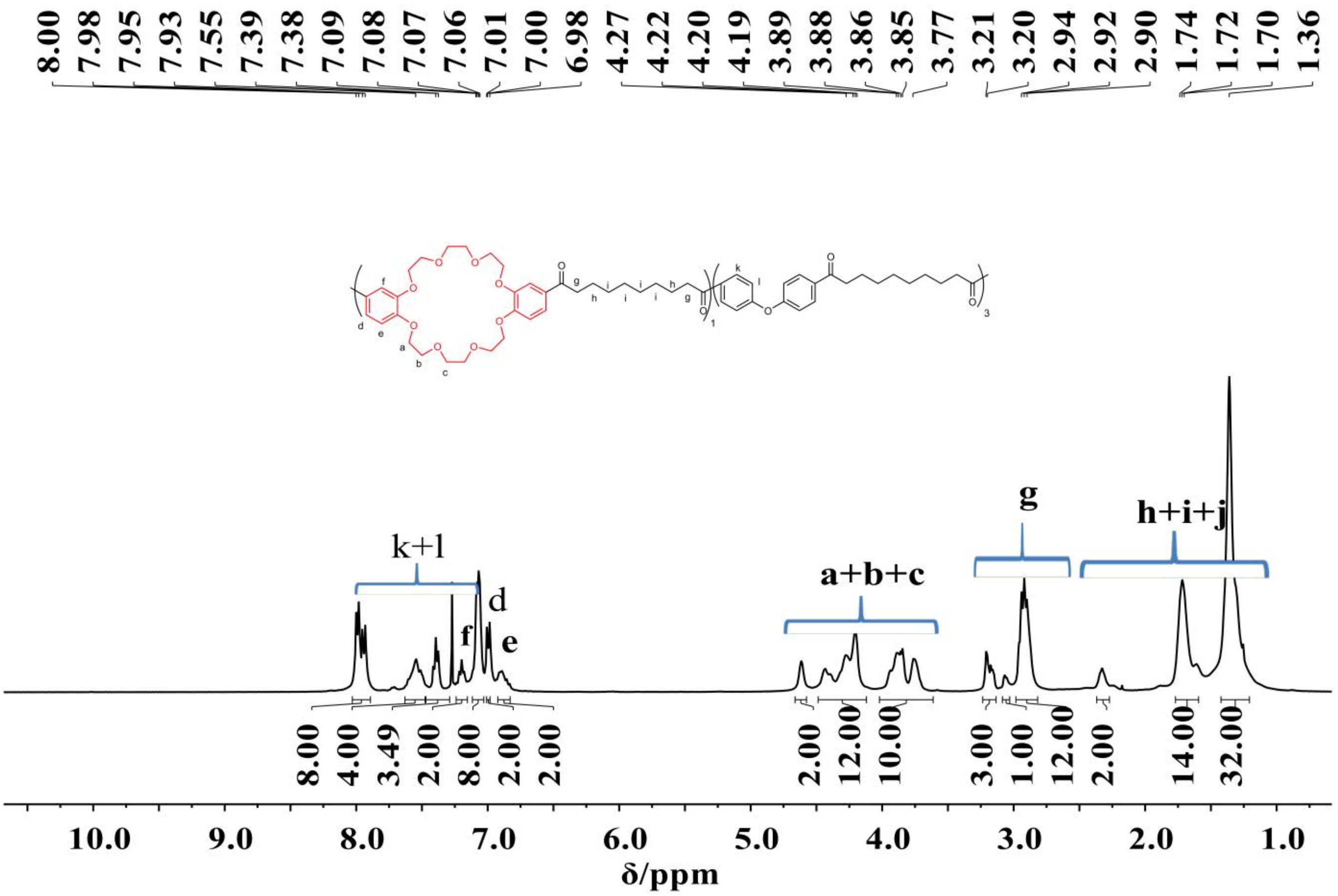

Supplementary Figure 2 ^{1}H NMR spectrum of **1** (400 MHz, $CDCl_3$, 298K).^{1}H NMR (400 MHz, $CDCl_3$, 298K) δ 7.97 (dd, J = 19.5, 8.1 Hz, 8H), 7.54 (td, J = 17.4, 16.3, 6.8 Hz, 4H), 7.40 (t, J = 7.9 Hz, 3H), 7.20 (t, J = 7.3 Hz, 2H), 7.07 (dd, J = 8.6, 3.8 Hz, 8H), 7.03-6.97 (m, 2H), 6.90 (dd, J = 8.8, 4.7 Hz, 2H), 4.74-4.56 (m, 2H), 4.40-4.12 (m, 12H), 3.94-3.70 (m, 10H), 3.20 (d, J = 4.4 Hz, 3H), 3.07 (t, J = 5.0 Hz, 1H), 3.03-2.79 (m, 12H), 2.33 (t, J = 7.4 Hz, 2H), 1.88-1.62 (m, 14H), 1.36 (s, 32H).

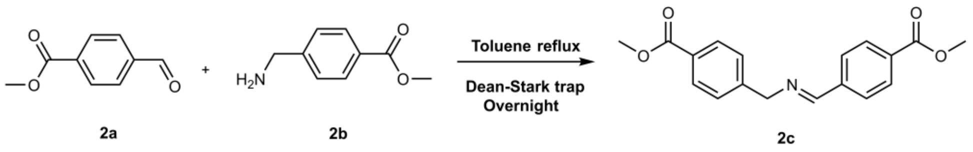

Supplementary Figure 3. Synthesis of **2c**.

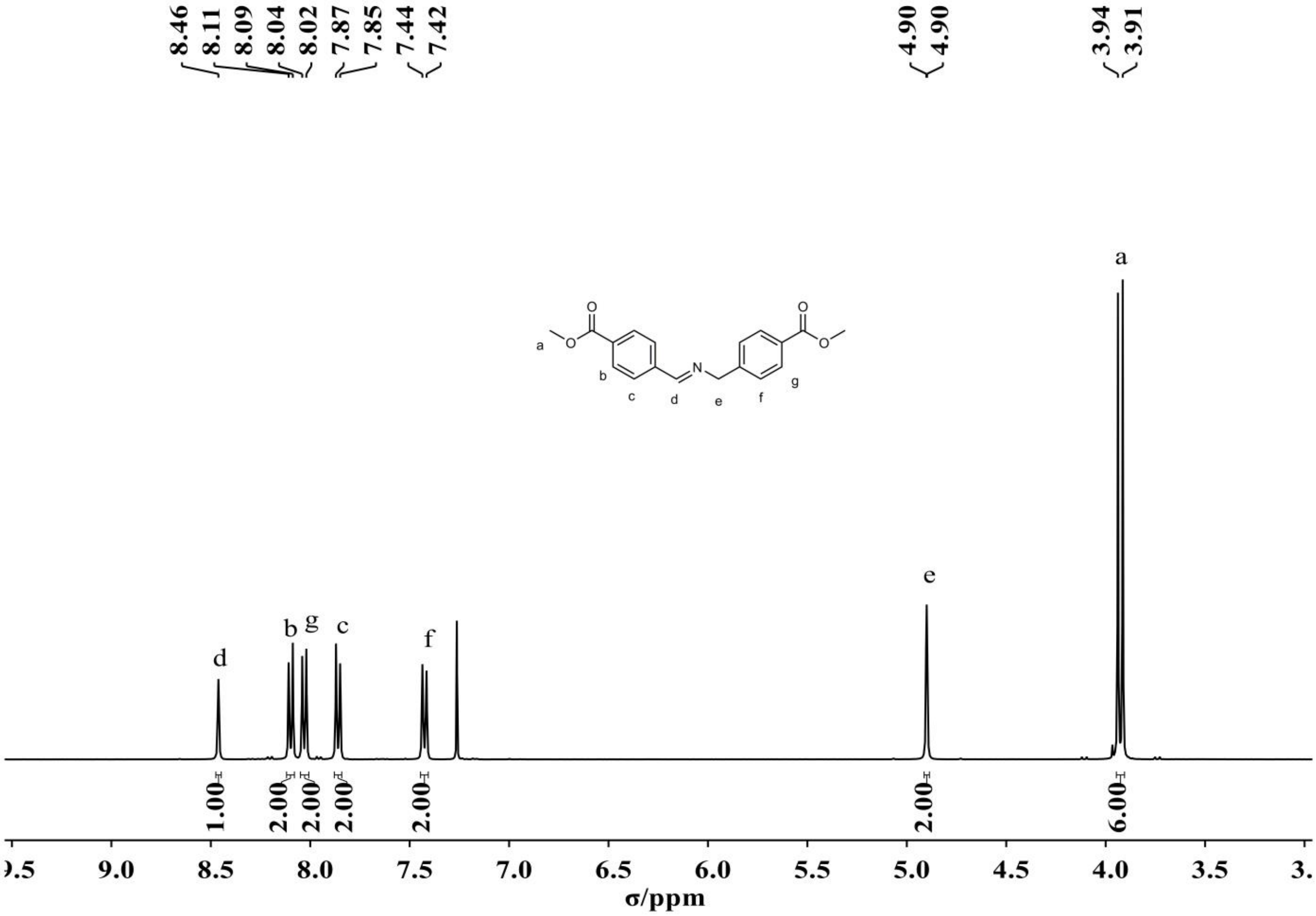

Supplementary Figure 4 ^{1}H NMR spectra of **2c (**400 MHz, $CDCl_3$, 298K). ^{1}H NMR (400 MHz, $CDCl_3$, 2.98K) δ 8.46 (d, J = 1.4 Hz, 1H), 8.10 (d, J = 8.4 Hz, 2H), 8.03 (d, J = 8.3 Hz, 2H), 7.86 (d, J = 8.4 Hz, 2H), 7.43 (d, J = 8.3 Hz, 2H), 4.90 (d, J = 1.3 Hz, 2H), 3.93 (d, J = 9.6 Hz, 6H).

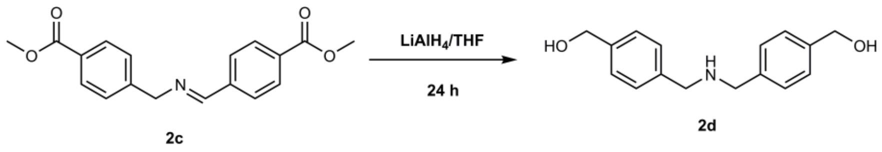

Supplementary Figure 5 Synthesis of **2d**.

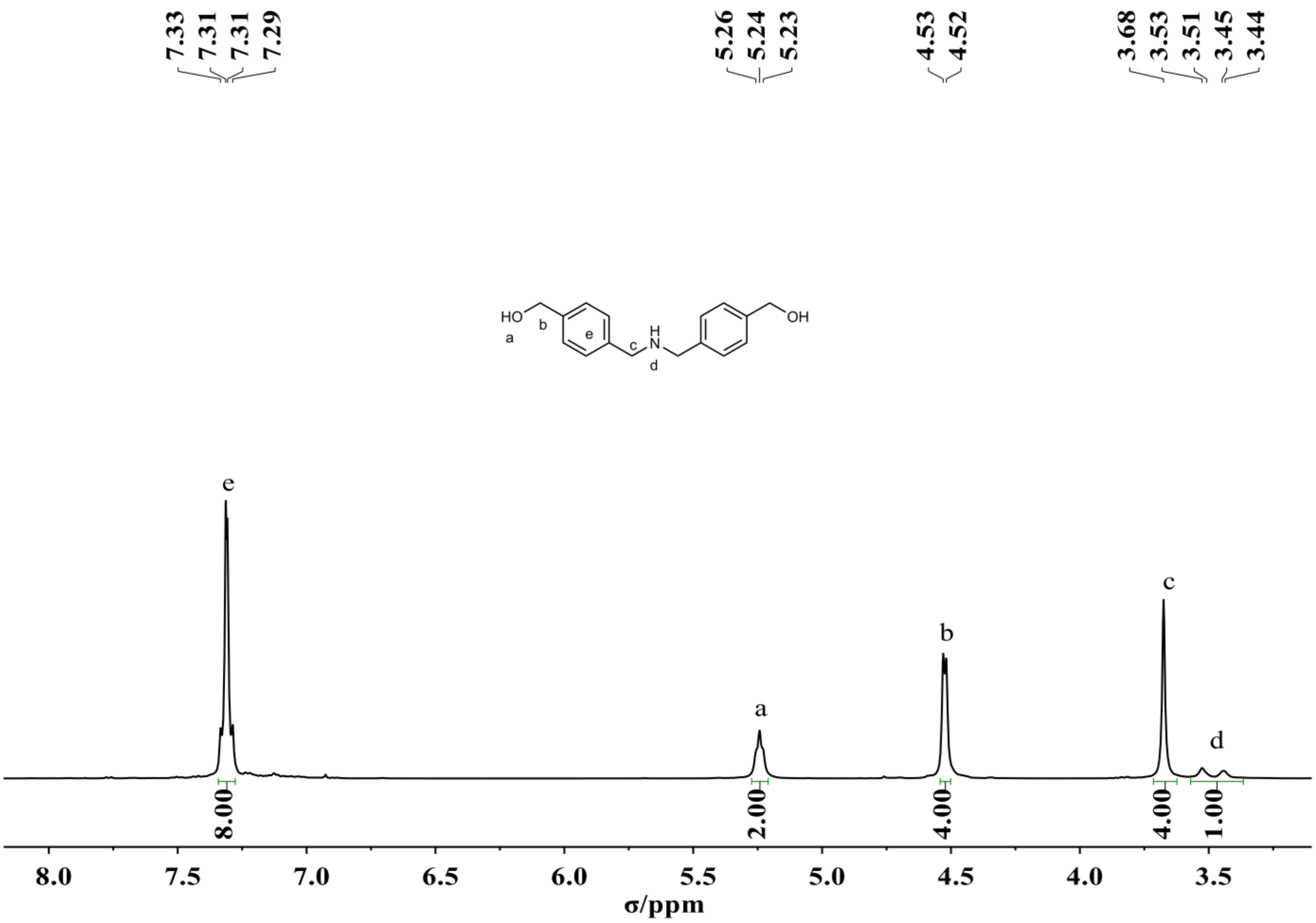

Supplementary Figure 6 ^{1}H NMR spectrum of **2d** (400 MHz, DMSO-d_6, 298K).^{1}H NMR (400 MHz, DMSO-d_6, 298K) δ 7.45-7.21 (m, 8H), 5.24 (t, J = 5.7 Hz, 2H), 4.52 (d, J = 4.8 Hz, 4H), 3.68 (s, 4H), 3.56-3.41 (m, 1H).

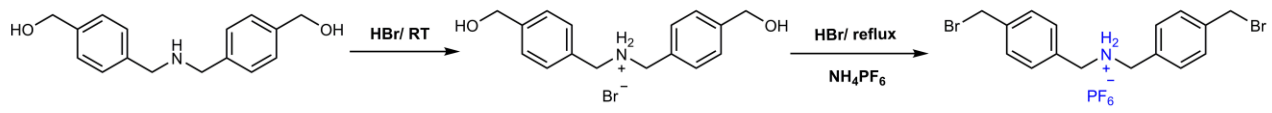

2d 2e 2

Supplementary Figure 7 Synthesis of **2**

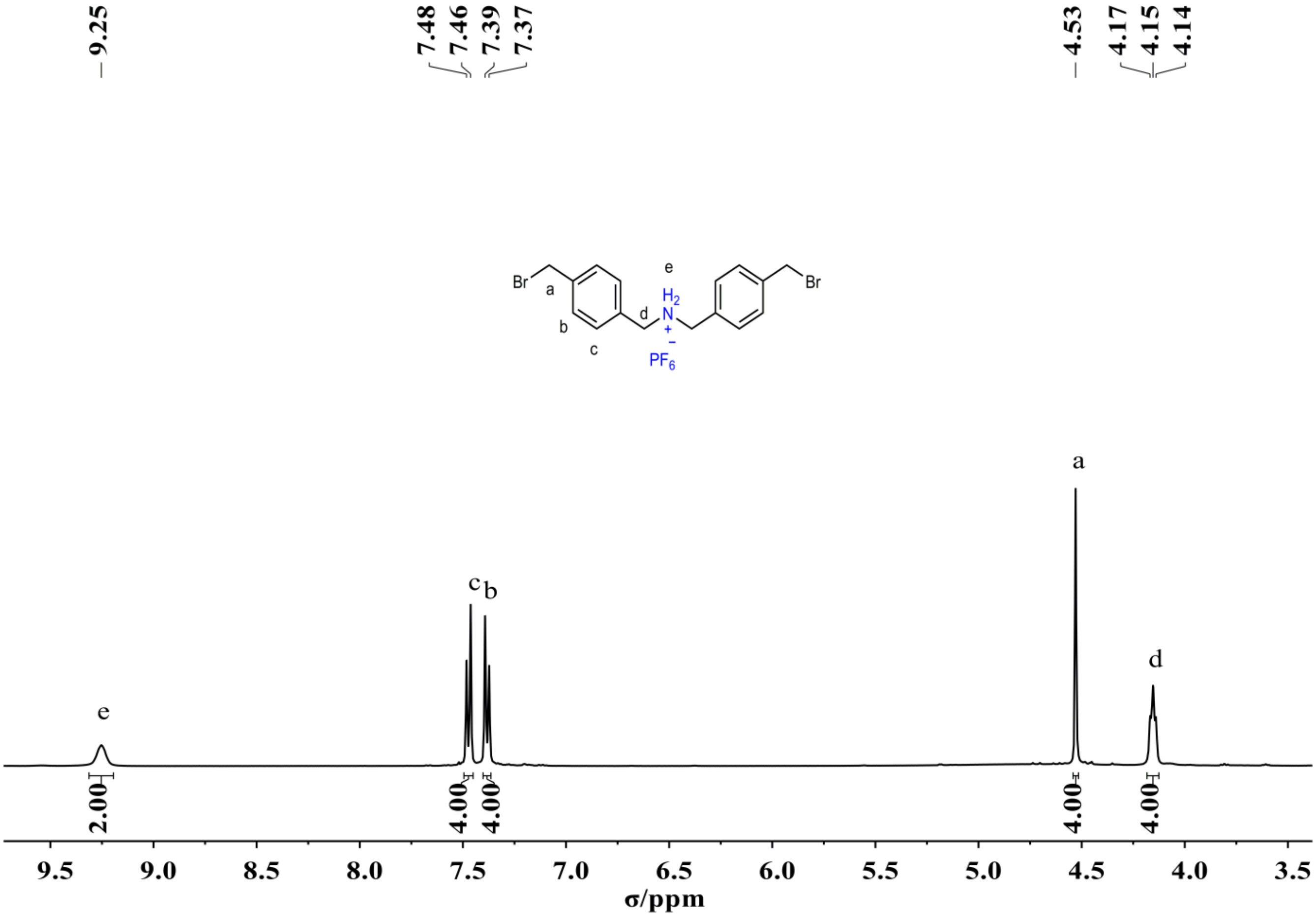

Supplementary Figure 8 ^{1}H NMR spectrum of **2** (400 MHz, DMSO-*d6*, 298K).^{1}H NMR (400 MHz, DMSO-d6, 298K) δ 9.25 (s, 2H), 7.47-7.38 (d, J = 7.9 Hz, 8H), 4.53 (s, 4H), 4.15 (t, J = 5.7 Hz, 4H).

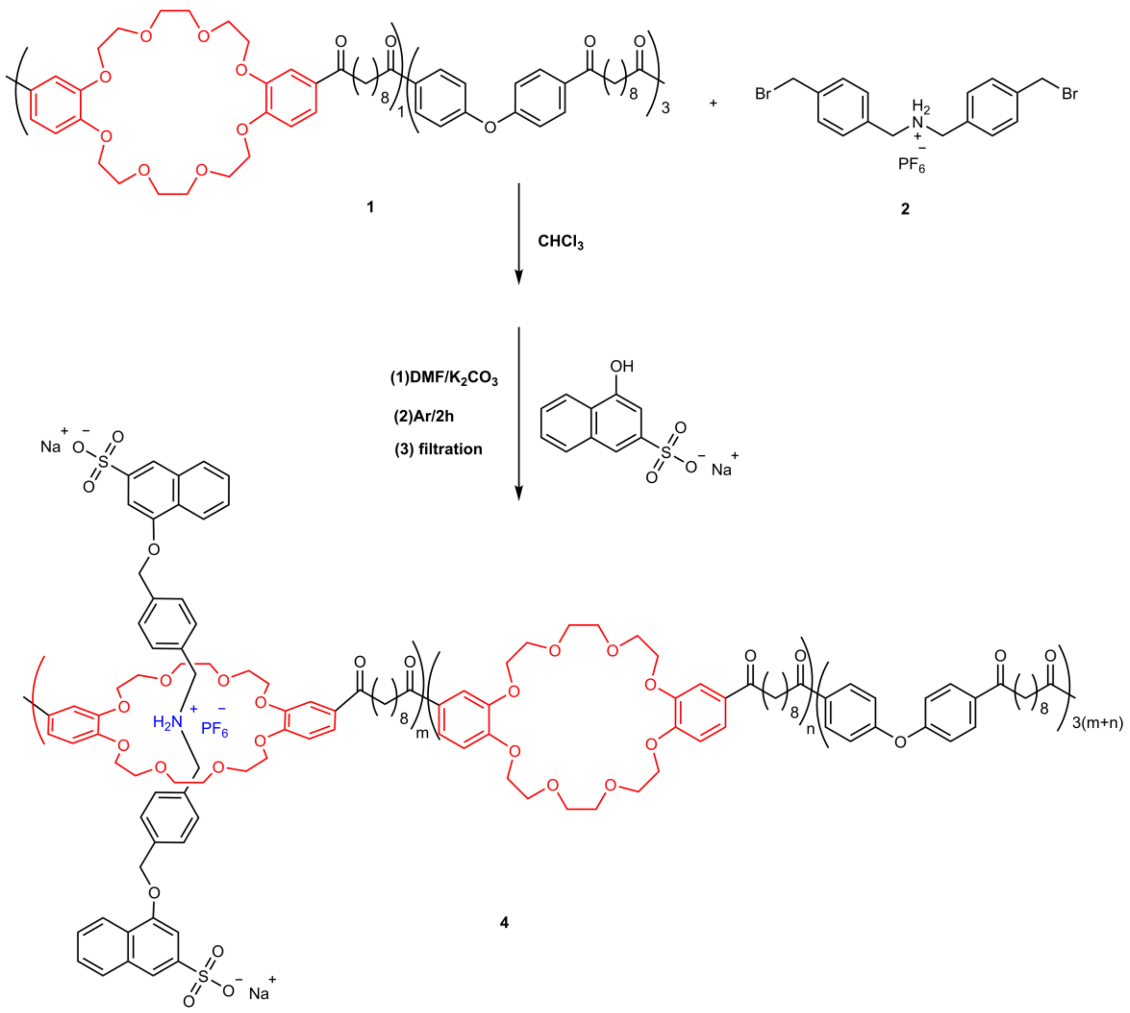

Supplementary Figure 9 Synthesis of **4**.

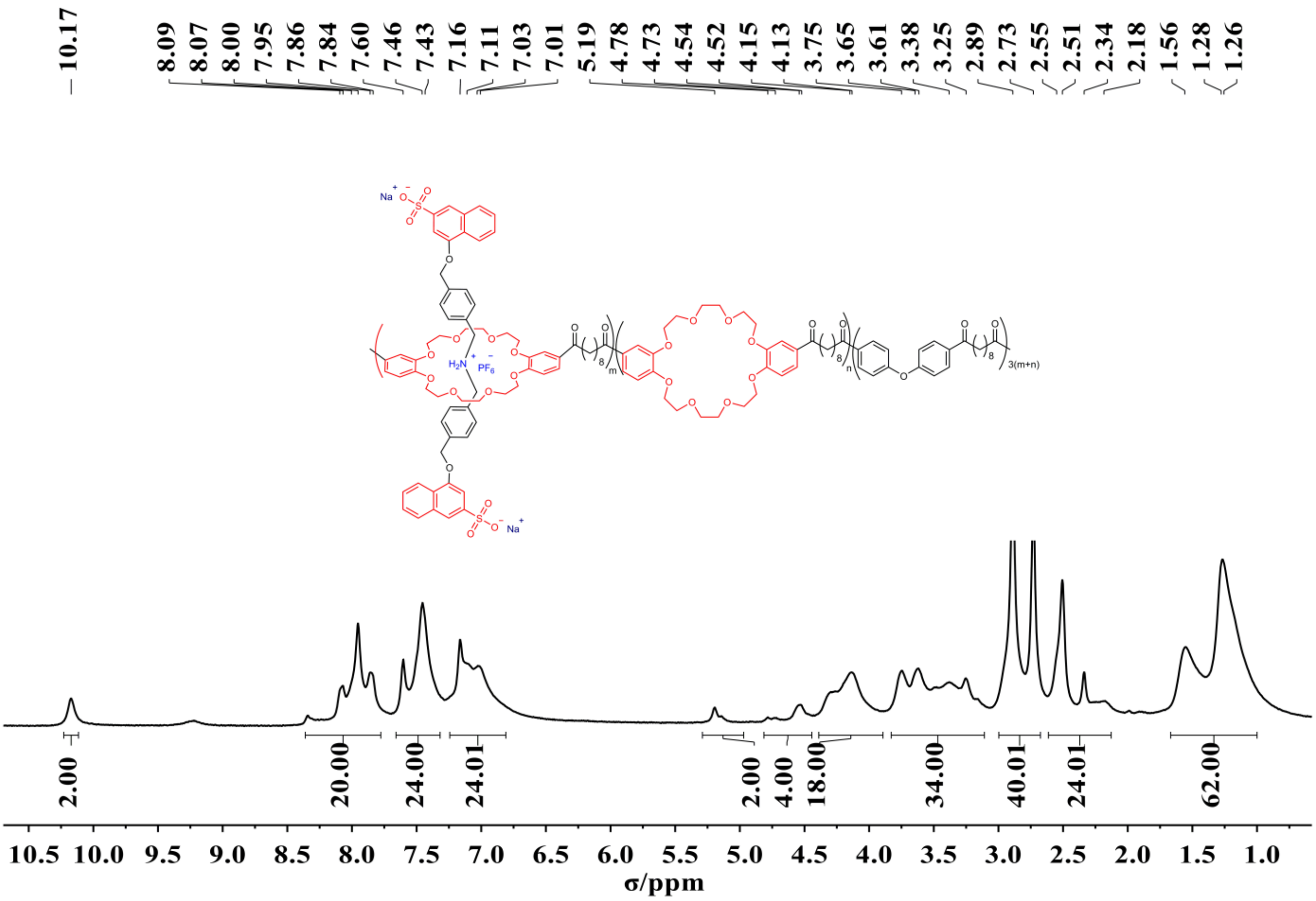

Supplementary Figure 10 ^{1}H NMR spectrum of **4** (300 MHz, DMSO-d_6, 298K).^{1}H NMR (300 MHz, DMSO-d_6, 298K) δ 10.17 (s, 2H), 8.26–7.74 (m, 20H), 7.71–7.29 (m, 24H), 7.28–6.89 (m, 24H), 5.19 (s, 2H), 4.66 (d, J = 72.9 Hz, 4H), 4.48–3.97 (m, 18H), 3.97–3.13 (m, 34H), 2.81 (d, J = 47.6 Hz, 40H), 2.56–2.28 (m, 24H), 1.41 (d, J = 90.9 Hz, 62H).

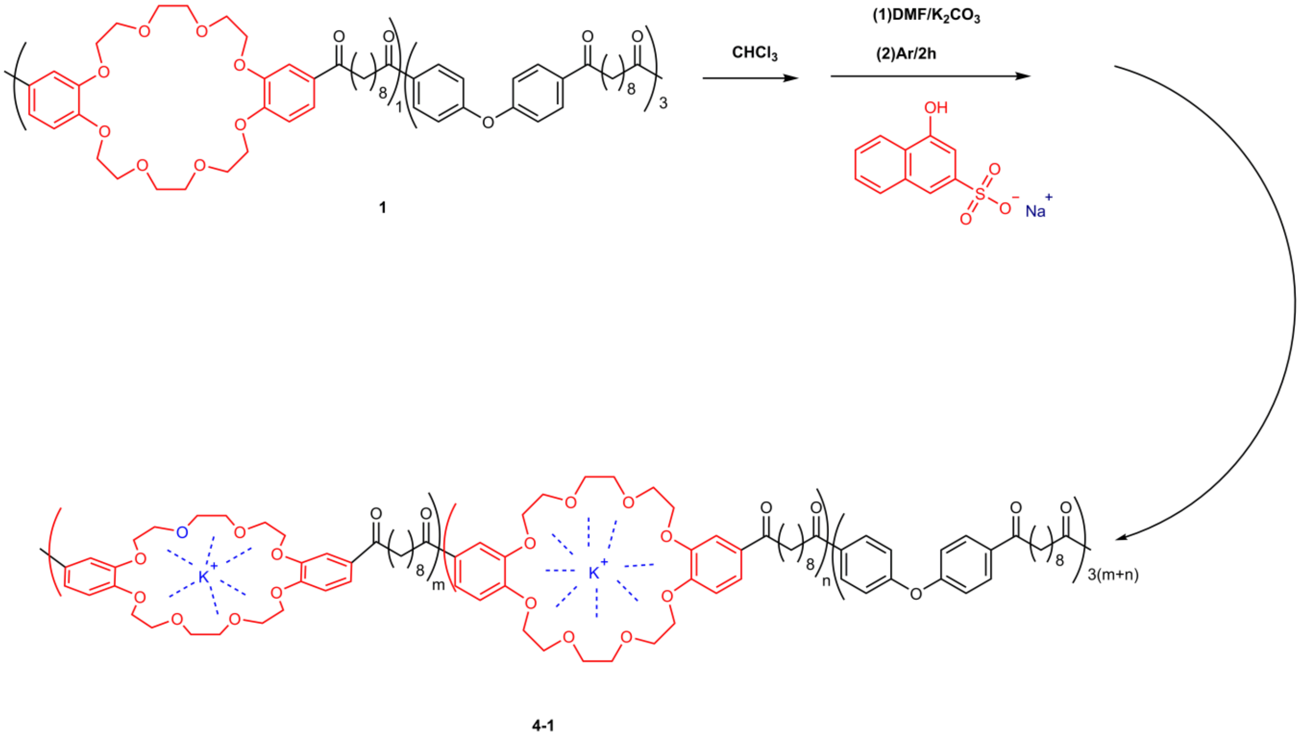

Supplementary Figure 11 Synthesis of **4-1**

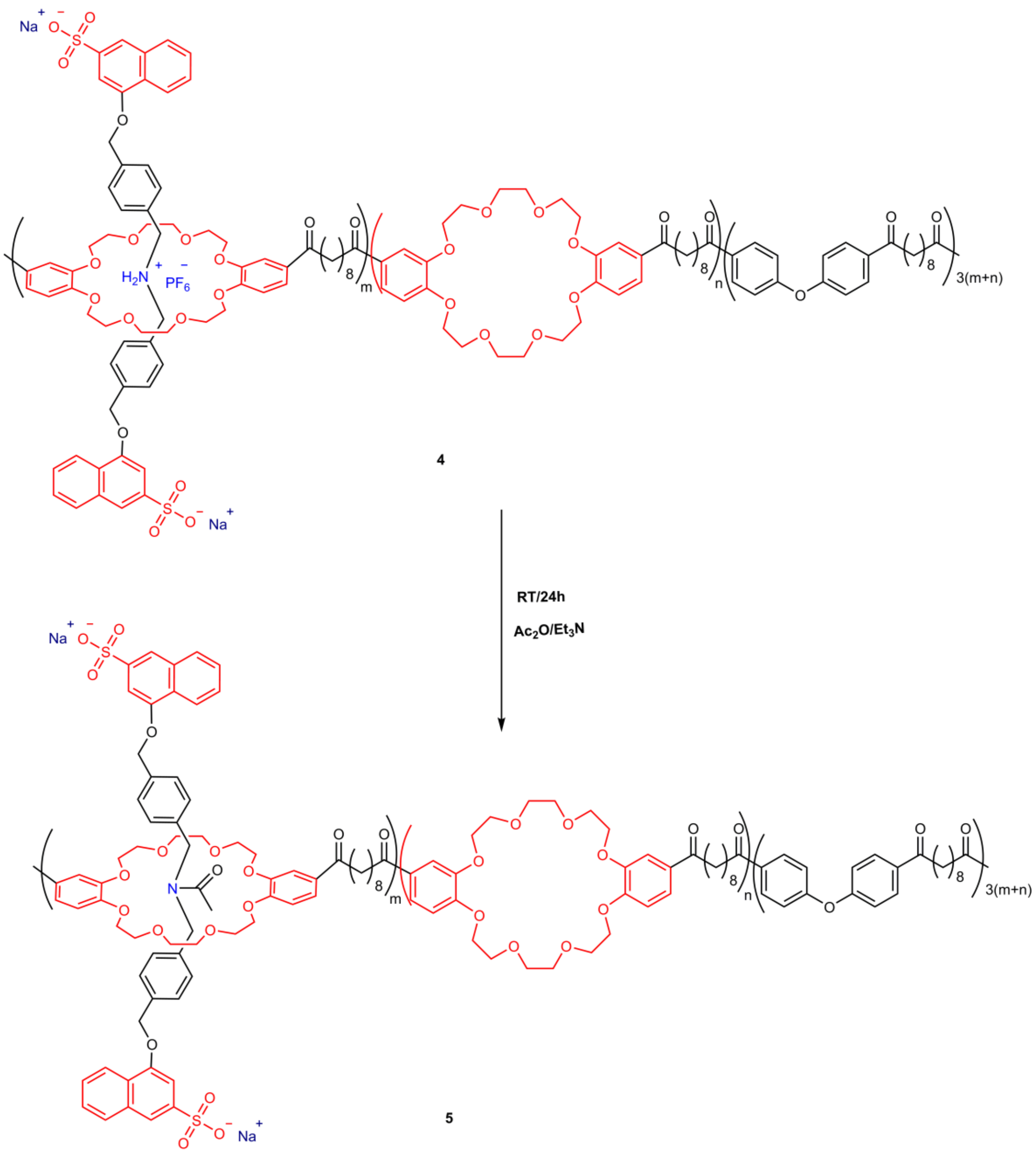

Supplementary Figure 12 Synthesis of **5**

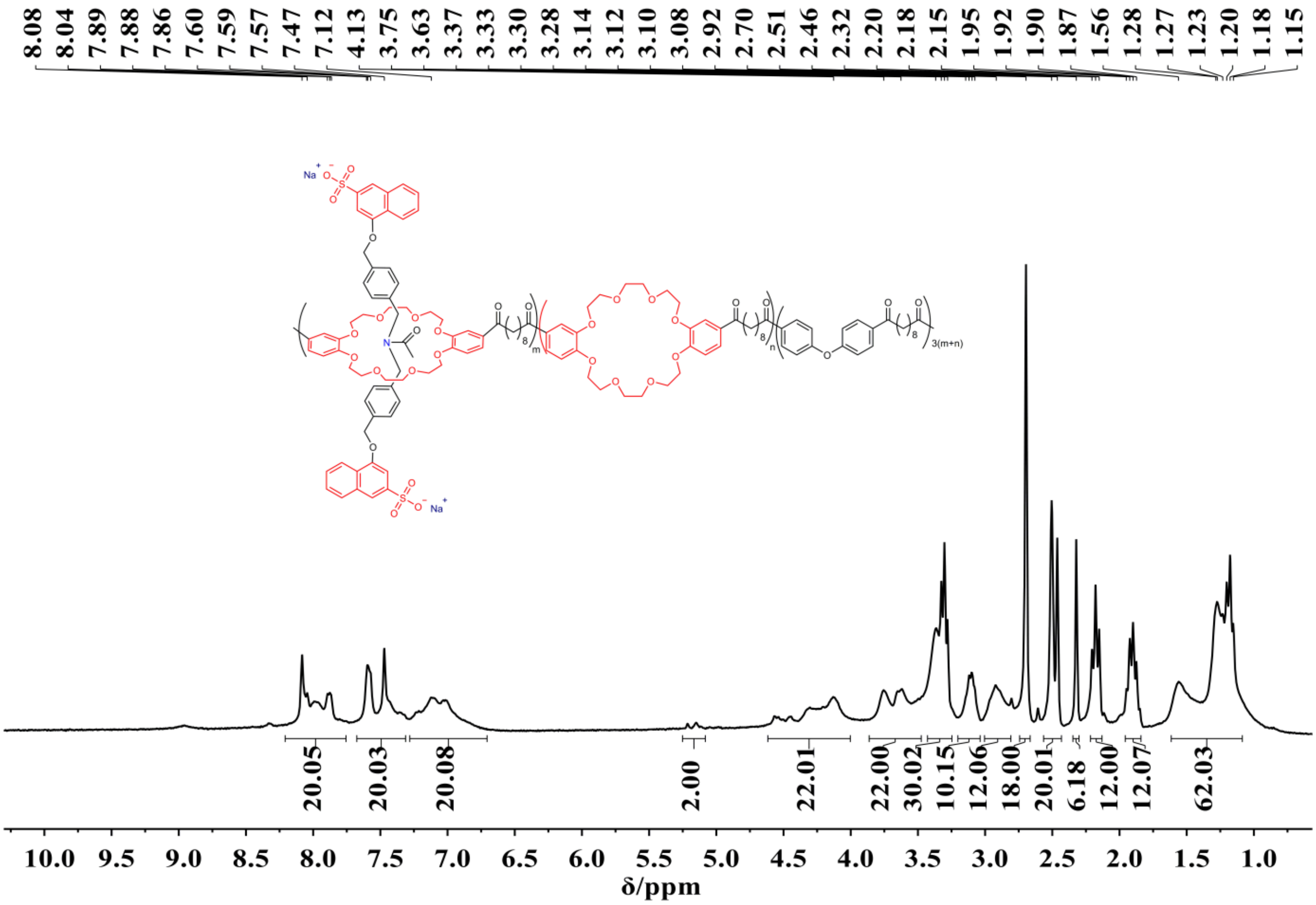

Supplementary Figure 13 ^{1}H NMR spectrum of **5** (400 MHz, DMSO-d_6, 298K).^{1}H NMR (400 MHz, DMSO-*d6*, 298K) δ 8.21–7.77 (m, 20H), 7.68–7.37 (m, 20H), 7.07 (d, J = 32.0 Hz, 20H), 5.18 (d, J = 18.6 Hz, 2H), 4.64–3.99 (m, 22H), 3.69 (d, J = 38.3 Hz, 22H), 3.45–3.23 (m, 30H), 3.11 (q, J = 6.8, 6.2 Hz, 10H), 2.92 (s, 12H), 2.70 (s, 18H), 2.48 (d, J = 12.7 Hz, 20H), 2.32 (s, 6H), 2.18 (t, J = 8.1 Hz, 12H), 1.91 (q, J = 7.6 Hz, 12H), 1.65–0.93 (m, 62H).

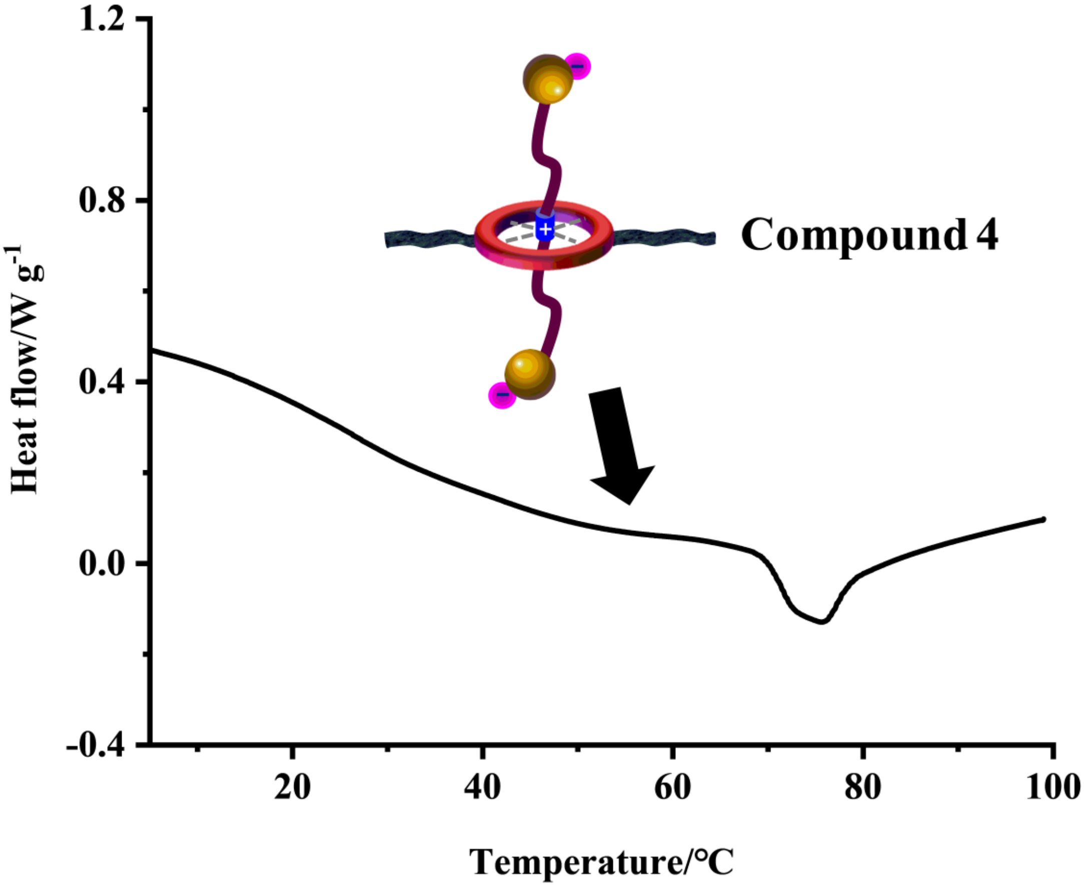

Supplementary Figure 14 DSC Curve of compound 4.

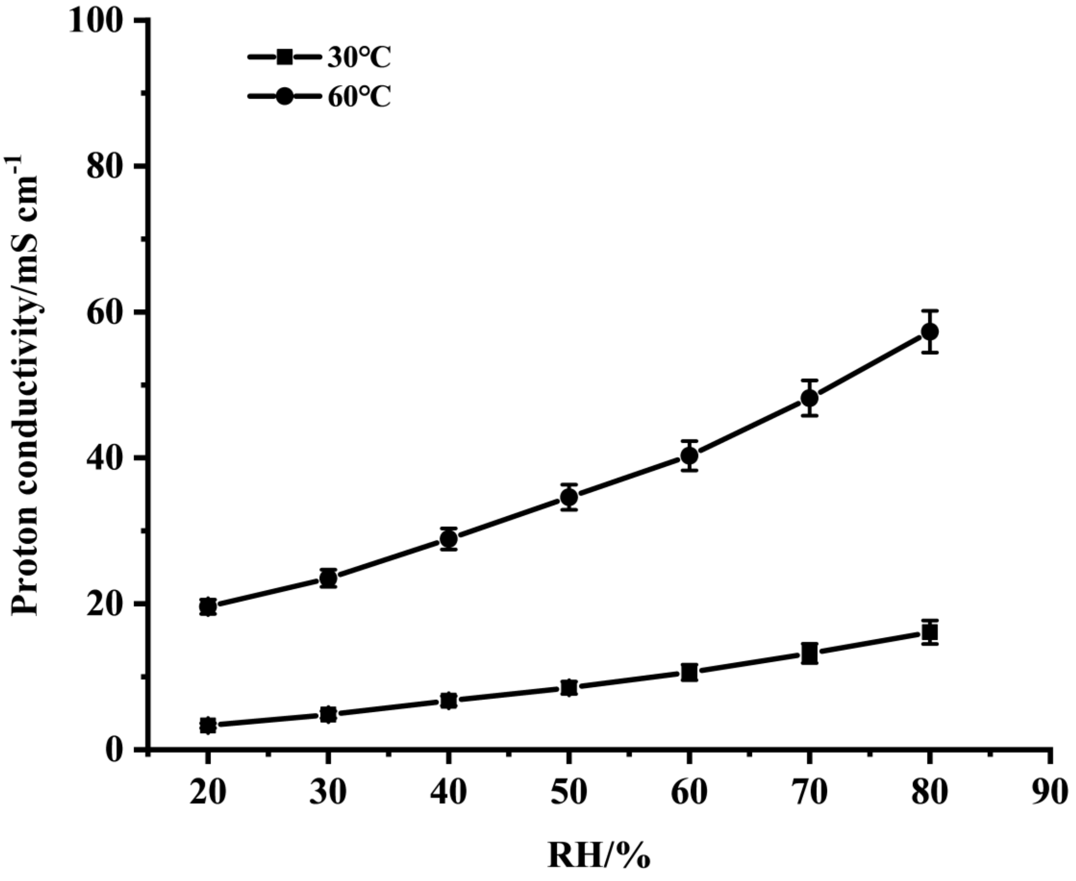

Supplementary Figure 15 Proton conductivity of the polyrotaxane membrane (IEC=0.73 mmol g^{-1}) as a function of membrane water vapor uptake at 30 ^{o}C and 60 ^{o}C. The error bars represent the s.d. from three independent measurements.

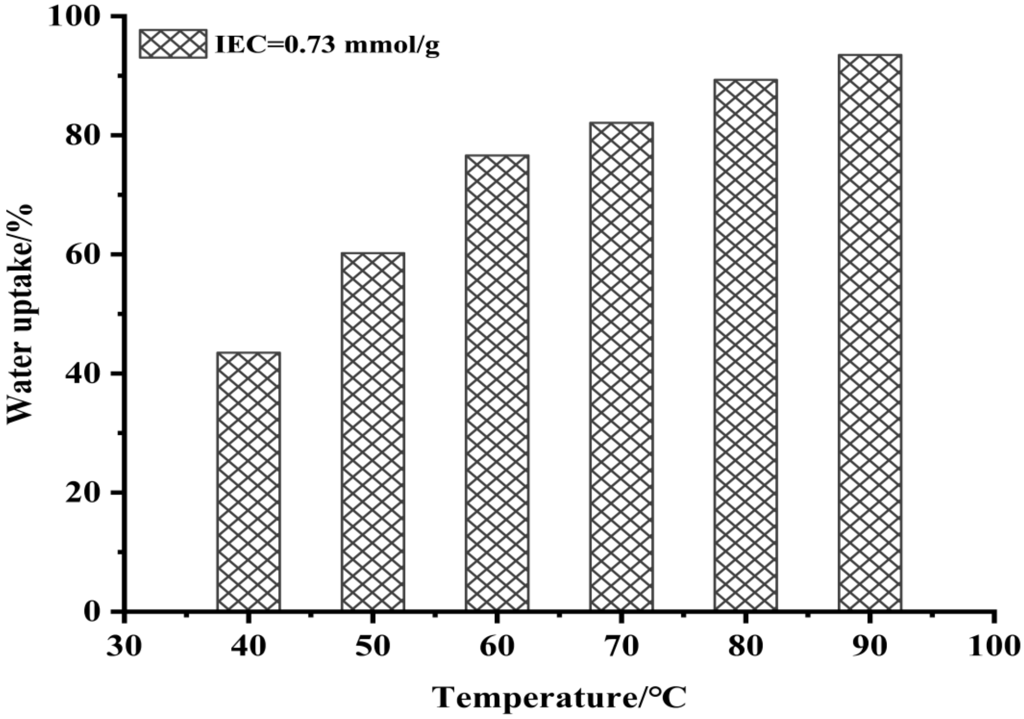

Supplementary Figure 16 Water uptake of the polyrotaxane membrane (IEC=0.73 mmol g^{-1}) as a function of temperature.

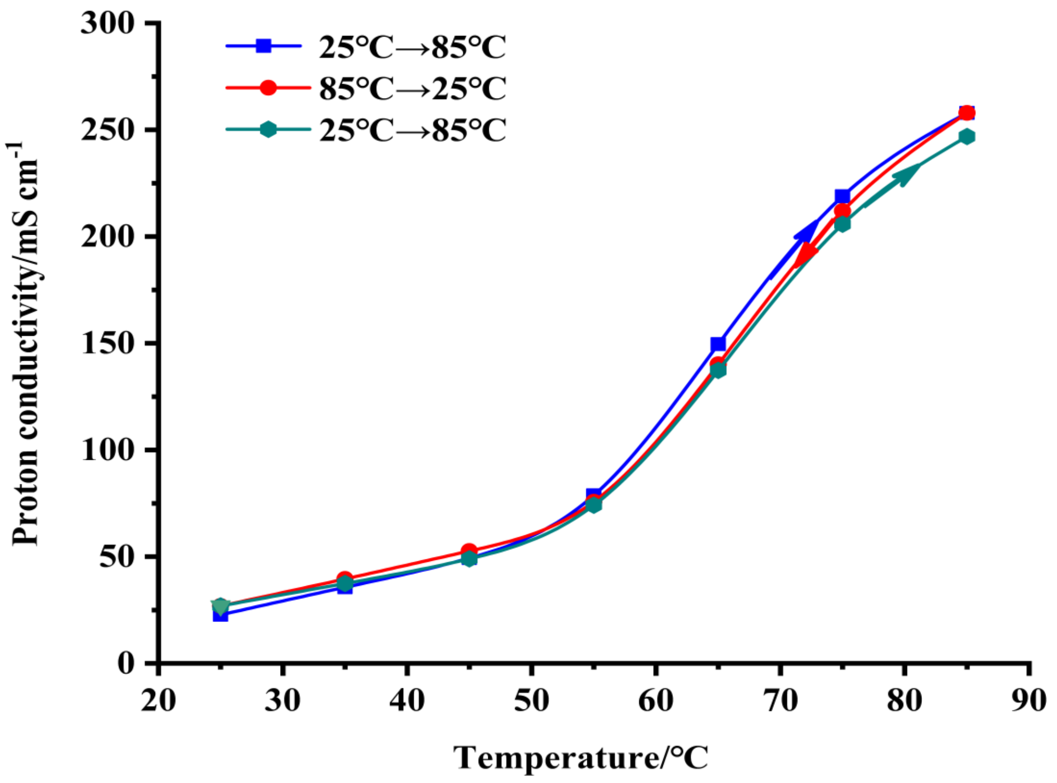

Supplementary Figure 17 The proton conductivity response of polyrotaxane membrane (IEC=0.73 mmol g^{-1}) to operation temperature change.

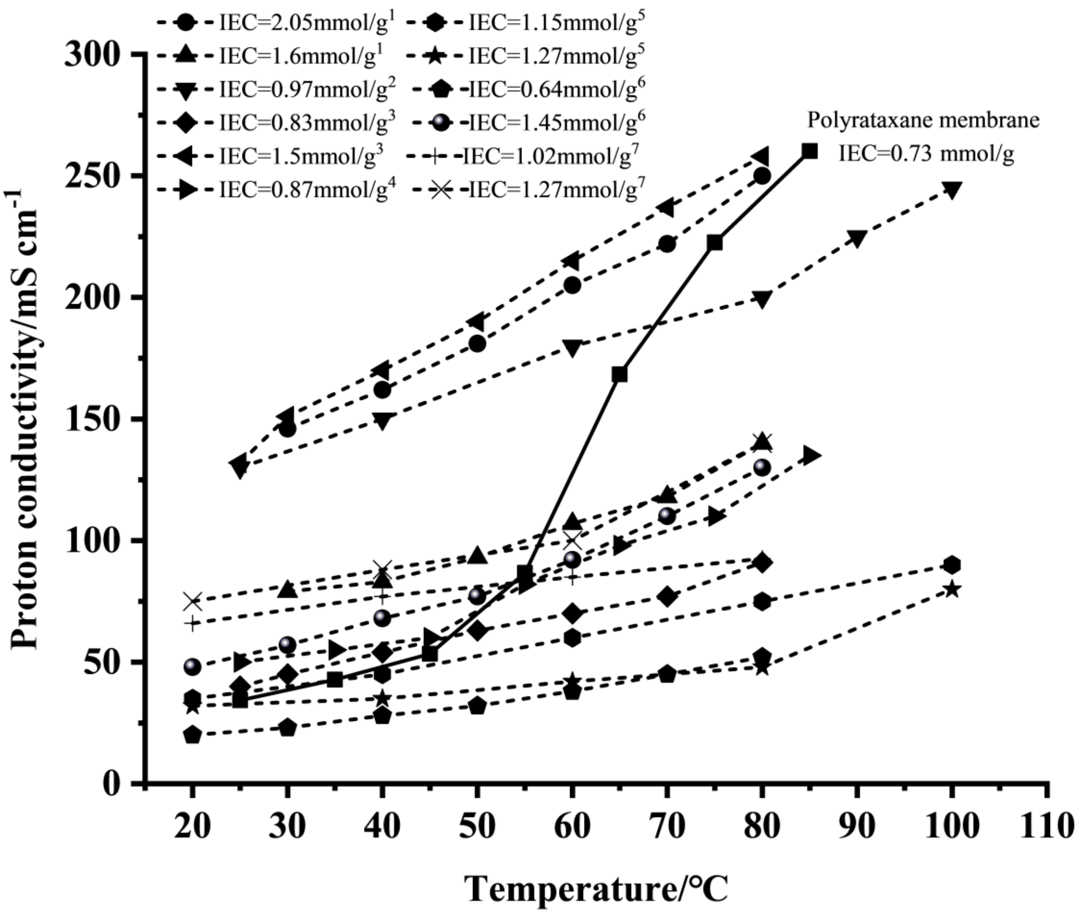

Supplementary Figure 18 Proton conductivities of the reported hydrocarbon-based proton exchange membranes (the dashed traces) and polyrotaxane membrane (the solid blue trace) as a function of temperature. [1-7]

Supplementary Table 1 Elemental analysis results for the polyrotaxane membrane

Name	Weight (mg)	N (%)	C (%)	S (%)	N Factor	C Factor	S Factor
polyrotaxane membrane	1.5880	1.22	58.68	2.336	0.9944	0.9865	1.0169

Supplementary Table 2 IEC values of polyrotaxane membrane samples from titration and elemental analysis

Membrane		Polyrotaxane # 1	Polyrotaxane # 2	Polyrotaxane # 3
IEC (mmol g^{-1})	Element analysis	0.25	0.42	0.73
	Titration	0.28	0.43	0.75

Supplementary Table 3 The tensile strength (TS) and elongation at break (Eb) of the polyrotaxane membrane (IEC=0.73 mmol g^{-1}) in the wet and dry states at 25 ^{o}C and 60 ^{o}C.

Membrane	25 ^{o}C		60 ^{o}C	
	Dry state	Wet state	Dry state	Wet state
TS (MPa)	0.10	9.60	1.86	12.01
Eb (%)	7.34	3.34	32.64	20.69

Supplementary References

1 Dong, W. S. *et al.* Durable Sulfonated Poly(arylene sulfide sulfone nitrile)s Containing Naphthalene Units for Direct Methanol Fuel Cells (DMFCs). *Macromolecules* **46**, 3452-3460 (2013).

2 Li, N., Lee, S. Y., Liu, Y.-L., Lee, Y. M. & Guiver, M. D. A new class of highly-conducting polymer electrolyte membranes: Aromatic ABA triblock copolymers. *Energy Environ. Sci.* **5**, 5346-5355(2012).

3 Zhou, Z. *et al.* Molded, high surface area polymer electrolyte membranes from cured liquid precursors. *J. Am. Chem. Soc.* **128**, 12963-12972(2006).

4 Norsten, T. B. *et al.* Highly Fluorinated Comb-Shaped Copolymers as Proton Exchange Membranes (PEMs): Improving PEM Properties Through Rational Design. *Adv. Funct. Mater.* **16**, 1814-1822 (2006).

5 Jutemar, E. P. & Jannasch, P. Locating sulfonic acid groups on various side chains to poly(arylene ether sulfone)s: Effects on the ionic clustering and properties of proton-exchange membranes. *J. Membr. Sci.* **351**, 87-95 (2010).

6 Lafitte, B. & Jannasch, P. Proton-Conducting Aromatic Polymers Carrying Hypersulfonated Side Chains for Fuel Cell Applications. *Adv. Funct. Mater.* **17**, 2823-2834 (2007).

7 Takamuku, S. & Jannasch, P. Multiblock Copolymers with Highly Sulfonated Blocks Containing Di- and Tetrasulfonated Arylene Sulfone Segments for Proton Exchange Membrane Fuel Cell Applications. *Adv. Energy Mater.* **2**, 129-140 (2012).
